# PRMT5 Prevents Cardiomyocyte Hypertrophy via Symmetric Dimethylating HoxA9 and Repressing HoxA9 Expression

**DOI:** 10.3389/fphar.2020.600627

**Published:** 2020-12-10

**Authors:** Sidong Cai, Rong Liu, Panxia Wang, Jingyan Li, Tingting Xie, Minghui Wang, Yanjun Cao, Zhuoming Li, Peiqing Liu

**Affiliations:** ^1^ Laboratory of Pharmacology and Toxicology, School of Pharmaceutical Sciences, National and Local United Engineering Lab of Druggability and New Drugs Evaluation, Guangdong Engineering Laboratoty of Druggability and New Drug Evaluation, Guangdong Provincial Key Laboratory of New Drug Design and Evaluation, Higher Education Mega Center, Sun Yat-Sen University, Guangzhou, China; ^2^ Obstetrical Department, Guangzhou Clifford Hospital, Guangzhou, China; ^3^ International Institute for Translational Chinese Medicine, School of Pharmaceutical Sciences, Guangzhou University of Chinese Medicine, Guangzhou, China; ^4^ School of Nursing, Guangdong Pharmaceutical University, Guangzhou, China

**Keywords:** protein arginine methyltransferase 5, homebox A9, cardiomyocyte hypertrophy, symmetric dimethylation, brain natriuretic peptide

## Abstract

The present study reveals a link between protein arginine methyltransferase 5 (PRMT5) and Homebox A9 (HoxA9) in the regulation of cardiomyocyte hypertrophy. In cardiomyocyte hypertrophy induced by β-adrenergic receptor agonist isoprenaline (ISO), PRMT5 expression was decreased while HoxA9 was upregulated. Silencing of PRMT5 or inhibition of PRMT5 by its pharmacological inhibitor EPZ augmented the expressions of cardiomyocyte hypertrophic genes brain natriuretic peptide (BNP) and β-Myosin Heavy Chain (β-MHC), whereas overexpression of PRMT5 inhibited ISO-induced cardiomyocyte hypertrophy, suggesting that PRMT5 ameliorates cardiomyocyte hypertrophy. On the contrary, HoxA9 promoted cardiomyocyte hypertrophy, as implied by the gain-of-function and loss-of-function experiments. HoxA9 was involved in the regulation of PRMT5 in cardiomyocyte hypertrophy, since HoxA9 knockdown prevented si-RPMT5-induced cardiomyocyte hypertrophy, and HoxA9 expression impaired the anti-hypertrophic effect of PRMT5. Co-immunoprecipitation experiments revealed that there were physical interactions between PRMT5 and HoxA9. The symmetric dimethylation level of HoxA9 was decreased by ISO or EPZ treatment, suggesting that HoxA9 is methylated by PRMT5. Additionally, PRMT5 repressed the expression of HoxA9. Chromatin immunoprecipitation (ChIP) assay demonstrated that HoxA9 could bind to the promoter of BNP, and that this binding affinity was further enhanced by ISO or EPZ. In conclusion, this study suggests that PRMT5 symmetric dimethylates HoxA9 and represses HoxA9 expression, thus impairing its binding to BNP promoter and ultimately protecting against cardiomyocyte hypertrophy. These findings provide a novel insight of the mechanism underlying the cardiac protective effect of PRMT5, and suggest potential therapeutic strategies of PRMT5 activation or HoxA9 inhibition in treatment of cardiac hypertrophy.

## Introduction

During cardiovascular diseases such as hypertension and atherosclerosis, cardiomyocytes are exposed to continuous mechanical stress and neurohumoral stimulation, resulting to reactivation of fetal genes such as atrial natriuretic factor (ANF), brain natriuretic peptide (BNP) and β-Myosin Heavy Chain (β-MHC), protein synthesis, sarcomere assembly and ultimately enhancement of cell surface area (Souders et al., 2009; Maillet et al., 2013; van Berlo et al., 2013; Oka et al., 2014; Lyon et al., 2015). This process contributes to the structural enlargement of the heart, namely pathological cardiac hypertrophy. Initially, cardiac hypertrophy is a compensation mechanism to maintain cardiac output. However, prolonged cardiac hypertrophy exacerbates the decrease of cardiac function, leading to arrhythmia, heart failure or sudden death. Accumulating epidemiological studies demonstrate the high incidence of cardiac hypertrophy worldwide, with the prevalence rate as about 1/500 to 1/200 (van Driel et al., 2019). Thus, it is essential to explore the molecular and cellular mechanisms underlying cardiac hypertrophy and to hunt for the therapeutic strategies.

PRMT5, referring to protein arginine methyltransferase 5, belongs to the histone methyltransferase PRMTs family that specifically transfers methyl groups from the methyl donor S-adenosine methionine (SAM) to the nitrogen atoms on the guanidium of arginine residues (Stopa et al., 2015; Kim et al., 2016). PRMT5 is regarded as the major type II arginine methyltransferase (Hadjikyriacou et al., 2015), which is mainly responsible for symmetric dimethylation on two different nitrogen atoms of the same guanidine residue from arginine substrates (Kim et al., 2016; Blanc and Richard, 2017). PRMT5 is able to methylate various histones and non-histone substrates, indicating its wide participation in biological process (Ren et al., 2010; Bandyopadhyay et al., 2012; Beltran-Alvarez et al., 2013; Tsai et al., 2013; Girardot et al., 2014; Yang et al., 2015; Webb et al., 2017). People with shrinking expression level of PRMT5 in peripheral blood are prone to developing stable coronary artery disease and acute myocardial infarction (Tan et al., 2019), suggesting that PRMT5 might play a critical role in cardiovascular diseases. Indeed, inhibition of PRMT5 facilitates cardiac hypertrophy through suppression of p300-mediated transcriptional activation of GATA4 (Chen et al., 2014).

The present study revealed that PRMT5 was able to regulate Homebox (HOX) A9 in cardiac hypertrophy. HOX genes encode a series of highly conservative evolutionary transcription factors harboring a DNA-binding motif of 60 amino acids (Tan et al., 2013). All of the 39 Hox genes are classified into four clusters, namely HoxA, HoxB, HoxC, HoxD respectively (Bhatlekar et al., 2014). Among the four clusters, HoxA cluster has attracted increasing attention because of its critical regulatory role in development of cardiovascular diseases, such as cardiac morphogenesis, ventricular septal defects, and endothelial inflammation (Di-Poi et al., 2010; Zhu et al., 2019a; Zhu et al., 2019b; Beachy et al., 2013; Makki and Capecchi, 2012). Recent studies have revealed that HoxA9, a member of HoxA cluster genes, was downregulated in hypertensive patients (Pirro et al., 2007), and that mRNA level of HoxA9 was enhanced in cardiac hypertrophy (Zhou et al., 2018).

Our preliminary studies showed that there were interactions between PRMT5 and HoxA9 in pathological cardiac hypertrophy and that HoxA9 was involved in the regulation of PRMT5 in cardiomyocyte hypertrophy. Thus, the present study was designed to investigate the underlying mechanisms.

## Materials and Methods

### Cell Culture

Neonatal rat cardiac myocytes (NRCMs) were collected from hearts of Sprague-Dawley (SD) rats one to three days after birth according to previous protocol (Golden et al., 2012). Hearts were instantly removed and washed in chilled phosphate-buffered saline (PBS), then hearts were chopped into small pieces. Next, small pieces were digested in 0.08% (w/v) trypsin solution at 37 °C for 5 min each time, totally 10–12 times. NRCMs were assembled by centrifuging at 1500 g for 6 min and re-suspended with DMEM (Dulbecco’s modified Eagle’s medium, Gibco, BRL Co., Ltd., USA) medium containing 10% fetal bovine serum (Cellmax, Beijing, China). After that, NRCMs were seeded in two 25 cm^2^ culture flasks for 1 h with 5% CO_2_ to separate cardiac fibroblasts and cardiomyocytes. Finally, cardiomyocytes were re-collected and seeded in culture dishes with 60–70% of confluent. To prevent potential contamination and fibroblasts proliferation, 1% penicillin-streptomycin and 0.1 mM 5-bromodeoxyuridine (5-BrdU) were necessary to added into the culture medium. 24 h later, culture medium was replaced by fresh one, and cells could be implied for subsequent experiments after another 12 h.

### Animals

Animal procedures used in this study were in line with institutional guidelines for the Care and Use of Laboratory Animals (NIH Publication No. 85-23, revised 1996) and were approved by Laboratory Animal Center of the Sun Yat-sen University. 12 Sprague-Dawley (SD) male rats of 200–220 g in SPF grade were obtained from the Experimental Animal Center of Sun Yat-sen University (Guangzhou, China). Briefly, SD rats were randomly allocated into two groups: the control (NS) group and isoprenaline (ISO) group. Rats in ISO group (n = 6) were intraperitoneally administrated with ISO (1.5 mg/kg/d, TargetMol), while rats in NS group received the same volume of saline. After 7-days-treatment, all rats were performed echocardiography by Vevo 2,100 ultrasound system (FUJIFILM VisualSonics Inc., Toronto, ON, Canada), in order to evaluate the left ventricular (LV) function. Subsequently, rats were anesthetized and sacrificed by intraperitoneally injecting 7% chloral hydrate. Hearts were immediately removed in diastolic state and trimmed transversely and separated in two parts. One part was fixed with 4% paraformaldehyde and embedded in paraffin for hematoxylin-eosin staining (HE staining), the other part was utilized for Western blotting analysis or Real-time qPCR assay.

### Antibodies and Reagents

The following primary antibodies were utilized in this study: Anti-HoxA9 (sc-81291), anti-αTubulin (sc-398103), anti-PRMT5 (sc-376937) and Anti-β-MHC (sc-53089) mouse monoclonal antibodies were purchased from Santa Cruz Biotechnology Inc. (Santa Cruz, CA, United States). Anti-BNP rabbit polyclonal antibody (PAA541Ra01) was purchased by Cloud-Clone Corp (Wuhan, Hubei, China). Anti-mouse Anti-mouse IgG, HRP-linked secondary antibody (#7076P2), and Anti-mouse Anti-rabbit IgG, HRP-linked secondary antibody (#7074P2) were purchased from Cell Signaling Technology, Inc. (Danvers, MA, United States).

ISO was acquired from TargetMol (Massachusetts, USA). Rhodamine phalloidin and 4′6-diamidino-2-phenylindole (DAPI) were purchased from YeasenBiotechCo. Ltd. (Shanghai, China). EPZ015666 was bought from MedChem Express (New Jersey, United States).

### Western Blotting (Hirano, 2012)

Total protein of NRCMs or heart tissues was extracted by RIPA lysis buffer (Beyotime, Nantong, Jiangsu, China). The concentration of protein samples were determined by BCA Protein Assay Kit (Thermo Fisher Scientific, MA, USA). Subsequently, equal amount of proteins from different samples, together with 5xSDS loading buffer (Beyotime, Nantong, Jiangsu, China) were loaded to 8∼12% sodium dodecyl sulfate-polyacrylamide gels and were subjected to electrophoresis (SDS-PAGE) under the condition of 70 V for 30 min and 110 V for 70 min. Then, proteins were transferred from gels to methanol-presoaked PVDF (polyvinylidene fluoride) membranes (EMD Millipore Corporation, Billerica, MA, USA) via under the constant current of 230 mA for 100 min. After that, membranes were blocked with 5% non-fat milk for 1 h and washed by TBST buffer (Tris-Buffered Saline Tween-20) for three times, 5 min each time. Membranes were incubated with primary antibodies at 4 °C overnight. After that, membranes were incubated with appropriate horseradish peroxidase (HRP)-conjugated secondary antibodies (Cell Signaling Technology, Danvers, MA) for another 1 h at ambient temperature. Blots were developed with enhanced chemiluminescence reagent (Pierce, Rockford, IL, United States) and detected by the LAS4000 imager (GE Healthcare, Waukesha, WI, United States). The intensities of the blots were quantified by the Quantity One (Bio-Rad) software. α-Tubulin was used as an internal control for total proteins.

### RNA Extraction and RT-PCR, Q-PCR

Total RNA from NRCMs or heart tissues were extracted by Trizol Reagent (Invitrogen, Carlsbad, CA, United States), according to manufacturer’s instructions. The concentration and purity of the RNA were measured by NanoDrop 2000 spectrophotometer from Thermo Fisher Scientific (Waltham, MA, USA). 2 μg of total RNA was reversed and transcripted into first strand cDNA by using RevertAid First Strand cDNA Synthesis Kit (Thermo Fisher Scientific) in RT-PCR system (Thermo Fisher Scientific). The mRNA expression levels were detected by loading cDNA, primer, buffer and THUNDERBIRD™ SYBR qPCR Mix (Toyobo, Osaka, Japan) in the proportion of 1:1:3:5 into one well of 96-well white plate and conducted in triplicate in PikoReal Real-Time PCR System (Thermo Fisher Scientific) for 40-cycle-amplilfication. Cycle values that reached threshold were recorded. mRNA expression was calculated by −2^∆∆CT^ method by using β-actin as internal control. Results were presented as fold change to control group. Primers of target genes were shown in Supplementary Material.

### Co-Immunoprecipitation

Experiments were conducted according to protocols (Smith et al., 2017). NRCMs or pre-chopped heart tissues were lysated by IP lysis buffer (Nantong, Jiangsu, China).

Totally 500 μg protein was used for immunoprecipitation, while additional 10 μg protein was used as input. In general, protein lysate was separately incubated with anti-PRMT5 antibody (Santa Cruz, CA, USA), anti-HoxA9 antibody (Santa Cruz, CA, USA) or anti-mouse IgG antibody (Beyotime, Nantong, Jiangsu, China) overnight at 4 °C. Then 25 μl of protein A/G beads (Thermo Fisher, New York, USA) was added to protein lysate and turned upside down at 4 °C. 4 h later, beads were washed, collected and boiled with 30 μl 2XSDS loading buffer, followed by SDS-PAGE immuno-blotting analysis.

### Measurement of Cell Surface Area

NRCMs cultured in 24-well plates were fixed with paraformaldehyde diluted in PBS buffer (4%, w/v) for 15 min at ambient temperature, followed by 0.3% Triton-100 treatment for 10 min. Then NRCMs were washed by PBS buffer. After that, NRCMs were incubated with 0.1% (v/v) rhodamine-phalloidin in dark for 1 h, and were further stained with DAPI before rinsing NRCMs for another three times with PBS buffer. Images of the NRCMs were detected via High Content Screening system (ArrayScanVTI, Thermo Fisher Scientific, Rockford, IL, United States). The cell surface area from randomly selected fields (3 for each group) was determined using ImageJ analysis software. Data was presented as fold change to control group.

### RNA Interference and Plasmid Transfection

Small interference RNAs (si-RNA) specific to PRMT5 (si-PRMT5) and HoxA9 (si-HoxA9), along with non-specific si-RNA (si-NC), were purchased from Guangzhou RiboBio Co., Ltd (Guangzhou, Guangdong, China). Target sequences were shown in Supplementary Material. Transfection was operated based on manufacturer’s instructions. Briefly, NRCMs seeded in 35 mm dishes with 60% confluent were transfected with 5 μl of Lipofectamine 2000 (Invitrogen, Carlsbad, CA, United States) and siRNA at final concentration of 20 μM in Opti-MEM® I Reduced-Serum Medium (Gibco, Grand Island, NY, USA). 6 h later, medium was replaced by fresh DMEM containing 10% of fetal bovine serum. NRCMs were incubated for additional 48–72 h before next step.

Wild type PRMT5 and mutant PRMT5 without methylation activity (mut-PRMT5, R367A, R368A) were both cloned into pcDNA3.1(+) vector by Vigenebio (Shandong, China). In general, 2 μg plasmid was transfected into cardiomyocytes in the same way of si-RNA transfection.

### Chromatin Immunoprecipitation Assay

Immunoprecipitated DNA was obtained by using SimpleChIP® Plus Enzymatic Chromatin IP Kit (#9005) from Cell Signaling Technology, Inc. Briefly, cells cultured in 100 mm plates with 80% of confluent were cross-linked with 1% of formaldehyde for 10 min, then cell pellets were sonicated and digested by 3 μl of micrococcal nuclease at 37 °C for 20 min to digest DNA to length of 150–900 bp. After that, cell pellets were immunoprecipitated with antibodies against PRMT5 or HoxA9, together with immunoglobulin G (IgG) antibody as negative control (Cell Signaling Technology) overnight. Subsequently, each immunoprecipitation sample was incubated with ChIP Grade Protein G Magnetic Beads contained in SimpleChIP® Plus Enzymatic Chromatin IP Kit for 2 h at 4 °C with rotation. DNA sample were eluted from Magnetic beads by using magnetic separation rack and proteinase K. DNA fragments were purified by using spin columns of the kit. Finally, purified DNA samples were applied for subsequent PCR assay to investigate the level of PRMT5 or HoxA9 binding to target DNA regions.

### Statistical Analysis

Data were presented as means ± SEM, and analyzed by two-tailed unpaired Student’s t-test between two groups and by one-way ANOVA followed by the *Bonferroni post hoc* test for multiple comparisons using GraphPad Prism Software Version 5.01 (La Jolla, CA). *p* < 0.05 was considered to be statistically significant.

## Results

### Homebox A9 Was Upregulated While Protein Arginine Methyltransferase 5 Was Downregulated in Isoprenaline-Induced Cardiomyocyte

**FIGURE 1:**
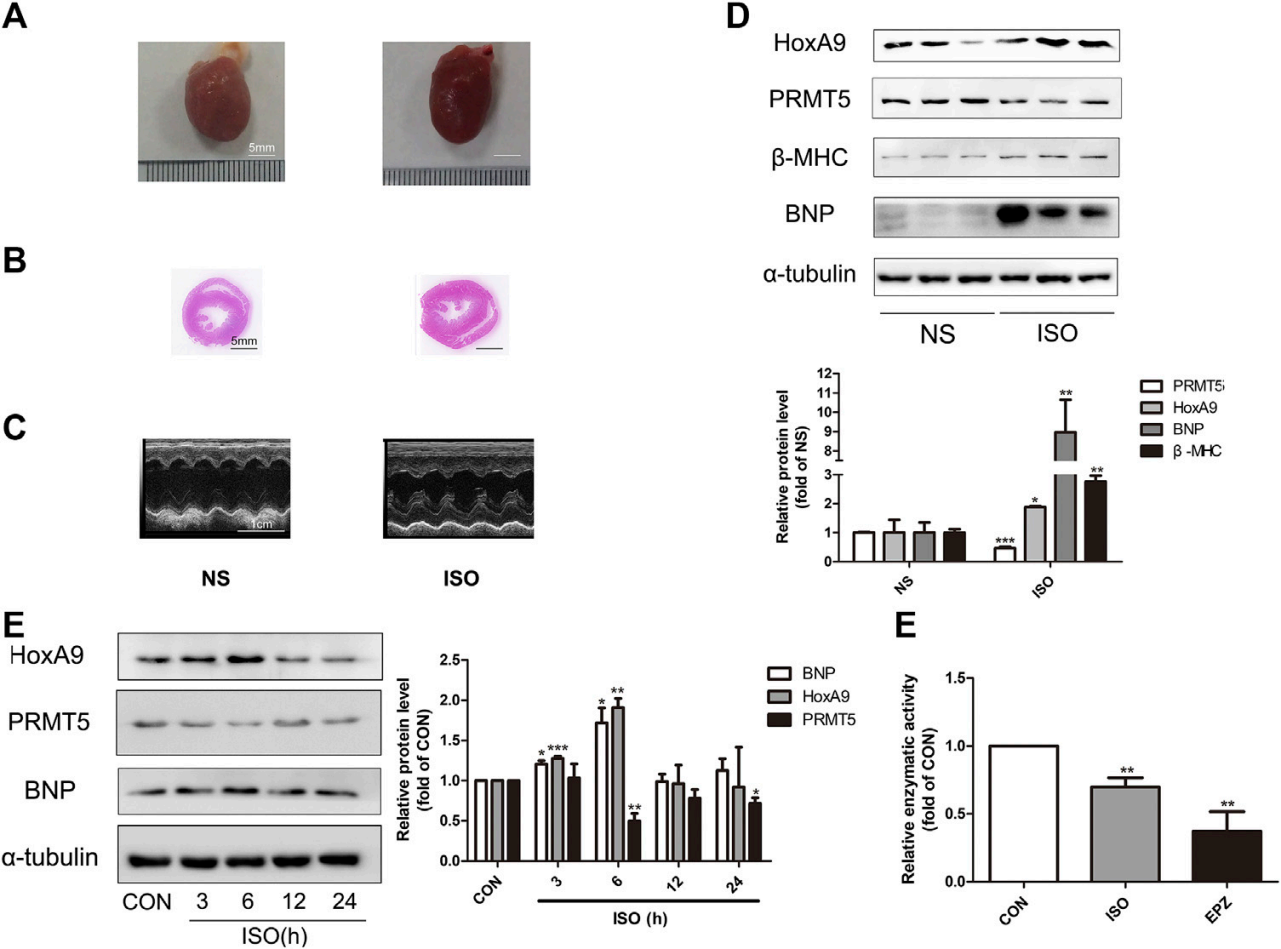
Homebox A9 was upregulated while protein arginine methyltransferase 5 was downregulated in isoprenaline-induced cardiac hypertrophy. **(A)** Representative gross morphology, **(B)** H&E staining of cross sections and **(C)** echocardiography for each group are as shown. Bar scale: 5 mm **(A–B)**. **(D)** Western blotting showing the expression level of Homebox A9 and hypertrophic protein BNP and β-Myosin Heavy Chain in cardiac tissues of rats treated with or without isoprenaline. Data are presented as mean ± SEM. *p < 0.05, **p < 0.01, as compared to control group. n = 6, samples were from six rats of each group. **(E)** Western blotting showing the expression level of Homebox A9, protein arginine methyltransferase 5 and brain natriuretic peptide in different time points in neonatal rat cardiomyocytes treated with or without isoprenalin. Data are presented as mean ± SEM. *p < 0.05, **p < 0.01, as compared to control group. n = 3, experiments were repeated 3 times.

Stimulation of β-adrenergic receptor by ISO could induce cardiac hypertrophy (Amoni et al., 2017; Schirmer et al., 2018). The expression level of HoxA9 and PRMT5 were investigated in a typical cardiac hypertrophic rat model induced by intraperitoneal administration of ISO for 7 days. As shown in ures 1A–Cures –ures C, the hearts of ISO-treated rats demonstrated larger morphological appearance, lower diameters of left ventricular cavity and thicker left ventricular wall, as compared to control rats. In addition, protein levels of hypertrophic markers BNP and β-MHC were significantly elevated in ISO group (Figure 1D). These data indicate that the *in vivo* cardiac hypertrophic model was successfully established. In this model, the protein expression of HoxA9 was found to be elevated, whereas PRMT5 was downregulated (Figure 1D).

*In vitroly*, NRCMs were treated with 10 μM ISO for different time points to induce cardiomyocyte hypertrophy. As demonstrated in Figure 1E, increased expression of HoxA9 and decreased level of PRMT5, along with the upregulation of BNP, were observed in a time-dependent manner. The results are in line with those *in vivo* data, confirming that HoxA9 was upregulated while PRMT5 was downregulated in ISO-induced cardiac hypertrophy.

Moreover, the enzymatic activity of PRMT5 was measured in cardiomyocytes treated with or without 10 μM ISO, or PRMT5 inhibitor 10 μM EPZ as a positive control. As shown in Figure 1F, the enzymatic activity of PRMT5 was significantly inhibited by ISO and EPZ, suggesting that the activity of PRMT5 is repressed in ISO-induced cardiomyocyte hypertrophy.

### Homebox A9 Promoted Cardiomyocyte Hypertrophy

**FIGURE 2:**
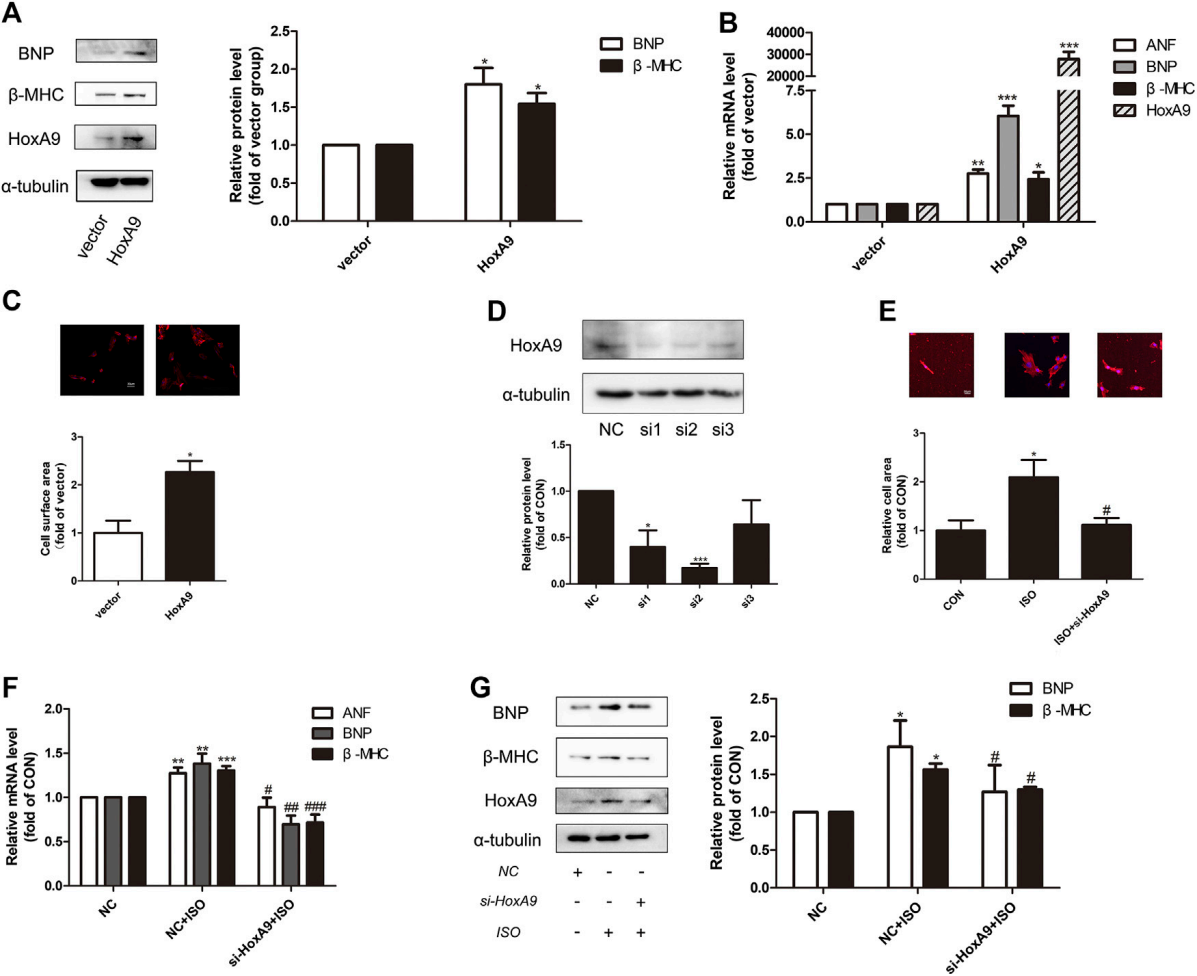
Homebox A9 evoked cardiac hypertrophy in Neonatal rat cardiac myocytes. **(A)** Western blotting showing the expression of cardiac hypertrophic marker brain natriuretic peptide and β-Myosin Heavy Chain in Neonatal rat cardiac myocytes transfected with vector plasmid or plasmid containing Homebox A9 DNA. Data are presented as mean ± SEM. *p < 0.05, **p < 0.01 as compared to vector group. n = 3, experiments were repeated 3 times. **(B–C)** RNA expression of atrial natriuretic factor, brain natriuretic peptide, β-Myosin Heavy Chain and cell surface area were shown in Neonatal rat cardiac myocytes transfected with vector or Homebox A9 DNA. Data are presented as mean ± SEM. *p < 0.05, **p < 0.01, ***p < 0.001 as compared to vector group. n = 3, experiments were repeated 3 times. **(D)** Western blotting showing the efficiency of small interference RNA target to Homebox A9. Data are presented as mean ± SEM. *p < 0.05, ***p < 0.001 as compared to NC group. n = 3. **(E–G)** Cell surface area, mRNA level of atrial natriuretic factor, brain natriuretic peptide, β-Myosin Heavy Chain and protein level of brain natriuretic peptide, β-Myosin Heavy Chain were shown in Neonatal rat cardiac myocytes transfected with NC, NC + isoprenalin, si-Homebox A9+isoprenalin group. Data are presented as mean ± SEM. *p < 0.05, **p < 0.01, ***p < 0.001 as compared to NC group, ^#^ *p* < 0.05, ^##^ *p* < 0.01, ^###^ *p* < 0.001 as compared to NC + isoprenalin group. n = 3, experiments were repeated 3 times.

Since the regulatory role of HoxA9 in cardiac hypertrophy was uncertain, gain-of-function and loss-of-function experiments were designed to investigate the effect of HoxA9 in NRCMs. Firstly, NRCMs were transfected with plasmid containing HoxA9 DNA. As shown in Figures 2A–C, the mRNA levels and protein levels of cardiomyocyte hypertrophic markers ANF, BNP and β-MHC, together with the cell surface area, were significantly augmented by HoxA9 overexpression. Secondly, small interference RNA was introduced to knock down endogenous HoxA9 in NRCMs. Si2-HoxA9, the most efficient siRNA, was selected for the subsequent studies (Figure 2D). HoxA9 silencing significantly reversed ISO-induced upregulation of hypertrophic markers and increase of cell surface area (Figures 2E–G). Taken together, these observations suggest that HoxA9 is able to promote cardiomyocyte hypertrophy, and that suppression of HoxA9 could protect against cardiomyocyte hypertrophy.

### Protein Arginine Methyltransferase 5 Ameliorated Cardiomyocyte Hypertrophy

**FIGURE 3:**
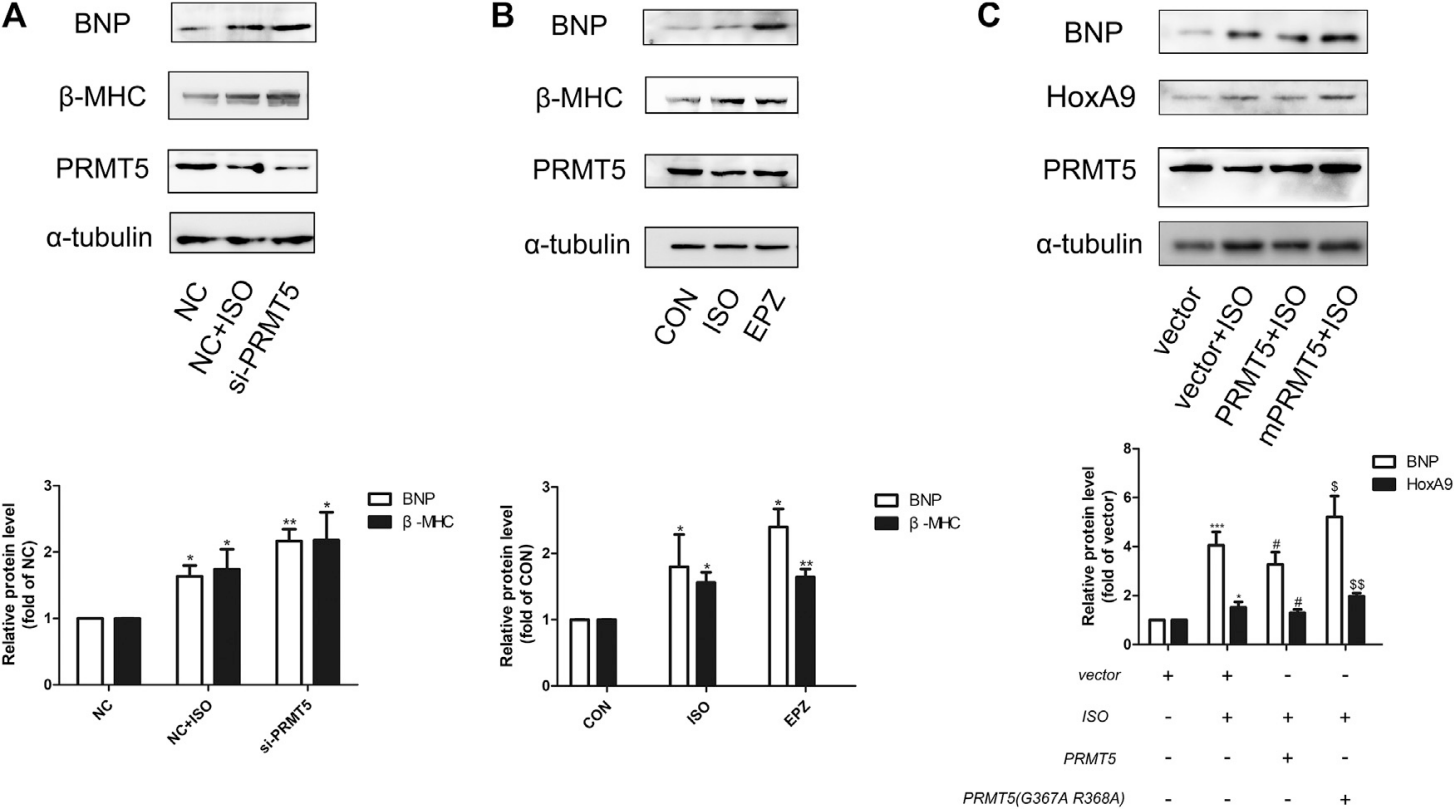
Protein arginine methyltransferase 5 was able to protect against cardiomyocyte hypertrophy. **(A)** Western blotting showing the expression of cardiac hypertrophic marker brain natriuretic peptide and β-Myosin Heavy Chain in Neonatal rat cardiac myocytes transfected with negative control (NC) in the presence or absence of isoprenalin, or protein arginine methyltransferase 5 small interference RNA (si-protein arginine methyltransferase 5) in the presence of isoprenalin. Data are presented as mean ± SEM. *p < 0.05, **p < 0.01 as compared to NC group. n = 3, experiments were repeated 3 times. **(B)** Western blotting showing the expression of brain natriuretic peptide and β-Myosin Heavy Chain in Neonatal rat cardiac myocytes treated with isoprenalin or protein arginine methyltransferase 5 enzymatic inhibitor, EPZ015666 (EPZ). Data are presented as mean ± SEM. *p < 0.05, **p < 0.01, ***p < 0.001 as compared to CON group, n = 3, experiments were repeated 3 times. **(C)** Western blotting showing the expression of brain natriuretic peptide and β-Myosin Heavy Chain in Neonatal rat cardiac myocytes transfected with vector in the presence or absence of isoprenalin, or protein arginine methyltransferase 5 DNA in the presence or absence of isoprenalin. Data are presented as mean ± SEM. *p < 0.05, ***p < 0.001 as compared to vector group, ^#^ *p* < 0.05 as compared to vector + isoprenalin group, ^$^ *p* < 0.05, ^$$^ *p* < 0.01 as compared to protein arginine methyltransferase 5 + isoprenalin group. n = 3, experiments were repeated 3 times.

The effect of PRMT5 in cardiomyocyte hypertrophy was examined. As demonstrated in Figures 3A,B, knockdown of PRMT5 or treatment with its enzymatic inhibitor EPZ (Chan-Penebre et al., 2015) obviously enhanced the expressions of hypertrophic markers BNP and β-MHC. Additionally, transfection of wildtype PRMT5 plasmid, but not the mutant PRMT5 without methylation activity, significantly reversed ISO-induced upregulation of hypertrophic marker BNP (Figure 3C). These results indicate that PRMT5 could ameliorate cardiomyocyte hypertrophy, and that this anti-hypertrophic effect is dependent on its methylation activity.

### Homebox A9 Was Involved in the Regulation of Protein Arginine Methyltransferase 5 in Cardiomyocyte Hypertrophy

**FIGURE 4:**
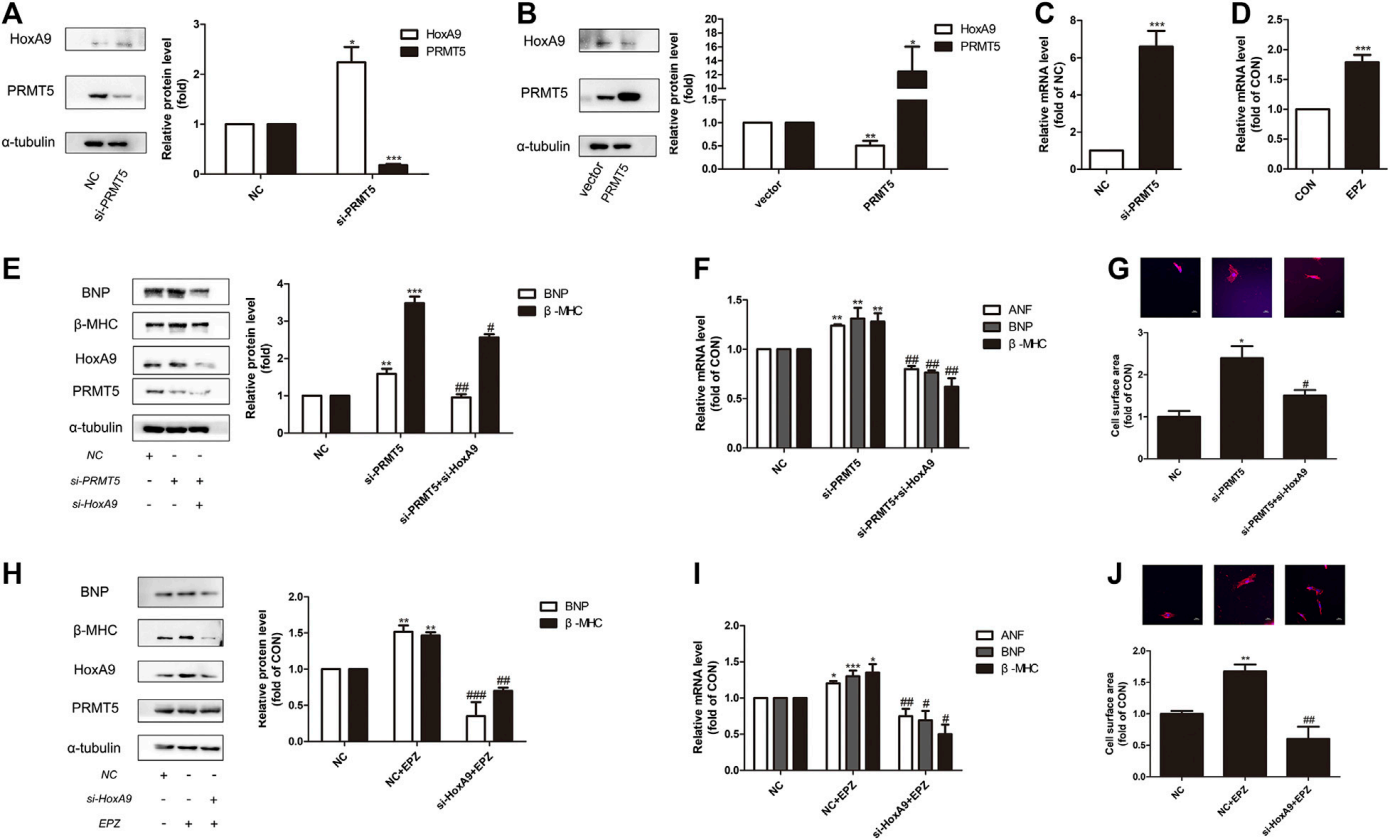
Homebox A9 suppression protected against cardiomyocyte hypertrophy induced by protein arginine methyltransferase 5 suppression or protein arginine methyltransferase 5 inhibition. **(A)** Western blotting showing the expression of Homebox A9 in Neonatal rat cardiac myocytes transfected with NC or si-protein arginine methyltransferase 5. Data are presented as mean ± SEM. *p < 0.05, ***p < 0.001 as compared to NC group. n = 3, experiments were repeated 3 times. **(B)** Western blotting showing the expression of Homebox A9 in Neonatal rat cardiac myocytes transfected with vector or protein arginine methyltransferase 5 DNA. Data are presented as mean ± SEM. *p < 0.05, **p < 0.01 as compared to vector group. n = 3, experiments were repeated 3 times. **(C)** Q-PCR assay demonstrating mRNA level of Homebox A9 of Neonatal rat cardiac myocytes transfected with NC or si-protein arginine methyltransferase 5. Data are presented as mean ± SEM. ***p < 0.001 as compared to NC group, n = 3, experiments were repeated 3 times. **(D)** Q-PCR assay demonstrating mRNA level of Homebox A9 of Neonatal rat cardiac myocytes treated with or without EPZ. Data are presented as mean ± SEM. ***p < 0.001 as compared to NC group, n = 3, experiments were repeated 3 times. **(E–G)** Protein level of brain natriuretic peptide, β-Myosin Heavy Chain, mRNA of atrial natriuretic factor, brain natriuretic peptide, β-Myosin Heavy Chain and cell surface area of Neonatal rat cardiac myocytes transfected with NC in the absence or presence of isoprenalin or si-protein arginine methyltransferase 5 in the presence of isoprenalin were shown. Data are presented as mean ± SEM. **p < 0.01, ***p < 0.001 as compared to NC group, ^#^ *p* < 0.05, ^##^ *p* < 0.01, ^###^ *p* < 0.001 as compared to si-protein arginine methyltransferase 5 group. n = 3, experiments were repeated 3 times. **(H–J)** Protein expression of brain natriuretic peptide, β-Myosin Heavy Chain, mRNA level of atrial natriuretic factor, brain natriuretic peptide, β-Myosin Heavy Chain and cell surface area in Neonatal rat cardiac myocytes transfected with NC in the absence or presence of EPZ, or transfected with si-Homebox A9 in the presence of EPZ were shown. Data are presented as mean ± SEM. *p < 0.05, **p < 0.01, ***p < 0.001 as compared to NC group, ^#^ *p* < 0.05, ^##^ *p* < 0.01, ^###^ *p* < 0.001 as compared to NC + EPZ group. n = 3, experiments were repeated 3 times.

Interestingly, it is found that HoxA9 participated in the anti-hypertrophic effect of PRMT5. As shown in Figures 4A,B, deficiency of PRMT5 augmented the protein expression of HoxA9, whereas overexpression of PRMT5 repressed HoxA9 expression. Consistently, silencing or inhibition of PRMT5 increased the mRNA expression of HoxA9 (Figures 4C,D).

Knockdown of HoxA9 prevented the increase of hypertrophic genes levels and cardiomyocyte size induced by si-PRMT5 (Figures 4E–G). Similarly, deficiency of HoxA9 reversed the hypertrophic effect of PRMT5 inhibitor EPZ (Figures 4H–J). Therefore, repression of HoxA9 is essential for the protective effect of PRMT5 against cardiomyocyte hypertrophy.

### Protein Arginine Methyltransferase 5 Could Interact With Homebox A9 and Symmetric Dimethylate Homebox A9

**FIGURE 5:**
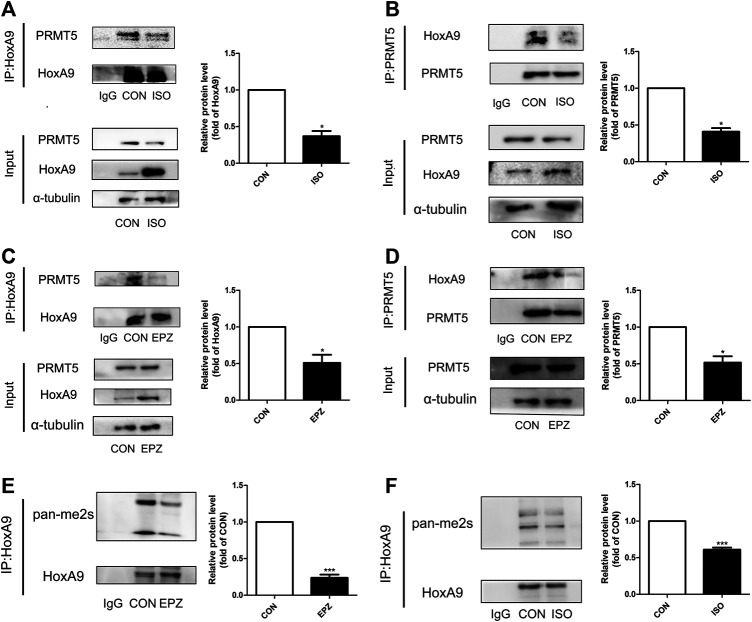
Protein arginine methyltransferase 5 could interact with Homebox A9 and symmetric dimethylate Homebox A9. **(A)** Co-immunoprecipitation assay showing the level of protein arginine methyltransferase 5 binding to Homebox A9 protein in Neonatal rat cardiac myocytes treated with or without isoprenalin. IgG served as negative control, data are presented as mean ± SEM. *p < 0.05 as compared to CON group. n = 3, experiments were repeated 3 times. **(B)** Co-immunoprecipitation assay showing the level of Homebox A9 binding to protein arginine methyltransferase 5 protein in Neonatal rat cardiac myocytes treated with or without isoprenalin. IgG served as negative control, data are presented as mean ± SEM. *p < 0.05 as compared to CON group. n = 3, experiments were repeated 3 times. **(C)** Co-immunoprecipitation assay showing the level of protein arginine methyltransferase 5 binding to Homebox A9 protein in Neonatal rat cardiac myocytes treated with or without EPZ. IgG served as negative control, data are presented as mean ± SEM. *p < 0.05 as compared to CON group. n = 3, experiments were repeated 3 times. **(D)** Co-immunoprecipitation assay showing the level of Homebox A9 binding to protein arginine methyltransferase 5 protein in Neonatal rat cardiac myocytes treated with or without EPZ. IgG served as negative control, data are presented as mean ± SEM. *p < 0.05 as compared to CON group. n = 3, experiments were repeated 3 times. **(E)** Co-immunoprecipitation assay showing the level of symmetric di-methylation level of Homebox A9 protein in Neonatal rat cardiac myocytes treated with or without EPZ. IgG served as negative control, data are presented as mean ± SEM. ***p < 0.001 as compared to CON group. n = 3, experiments were repeated 3 times. **(F)** Co-immunoprecipitation assay showing the level of symmetric di-methylation level of Homebox A9 protein in Neonatal rat cardiac myocytes treated with or without isoprenalin. IgG served as negative control, data are presented as mean ± SEM. ***p < 0.001 as compared to CON group. n = 3, experiments were repeated 3 times.

Co-immunoprecipitation results showed that there were physical interactions between PRMT5 and HoxA9 (Figures 5A,B). During ISO-induced cardiomyocyte hypertrophy, the interactions of PRMT5 and HoxA9 were suppressed (Figures 5A,B). Similarly, treatment of EPZ abrogated these interactions (Figures 5C,D), suggesting that the interactions between these two molecules are probably dependent on the enzymatic activity of PRMT5. Since PRMT5 is a principle symmetric dimethylase, it is hypothesized that PRMT5 might directly symmetric dimethylate HoxA9. To test this hypothesis, the symmetric dimethylation level of HoxA9 was determined. As shown in Figures 5E,F, the symmetric dimethylation level of HoxA9 was decreased by ISO or EPZ treatment. Thus, these observations suggest that PRMT5 could interact with and symmetric dimethylate HoxA9. When PRMT5 was suppressed in cardiomyocyte hypertrophy, or inhibited by EPZ, the symmetric dimethylation of HoxA9 would be weakened.

### Repression of Protein Arginine Methyltransferase 5 Facilitated Homebox A9 Binding to Brain Natriuretic Peptide Promoter

**FIGURE 6:**
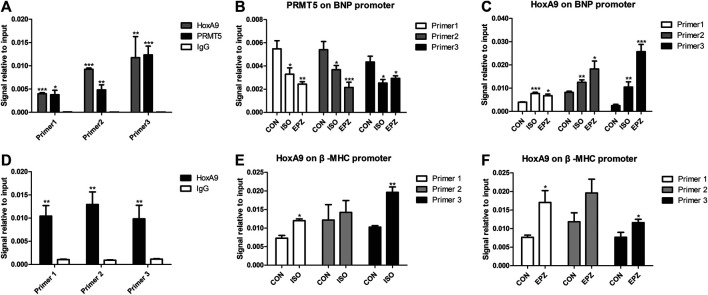
Binding of protein arginine methyltransferase 5 to promoter regions of brain natriuretic peptide gene was decreased while binding of Homebox A9 to brain natriuretic peptide promoter was increased in cardiomyocyte hypertrophy induced by isoprenalin or EPZ. **(A)** ChIP-qPCR assay showing the binding ability of Homebox A9, protein arginine methyltransferase 5 and IgG (negative control) on three promoter regions of brain natriuretic peptide gene. Data are presented as mean ± SEM. *p < 0.05, **p < 0.01, ***p < 0.001 as compared to IgG group. n = 3, experiments were repeated 3 times. **(B)** ChIP-qPCR assay showing the binding ability of protein arginine methyltransferase 5 on three promoter regions of brain natriuretic peptide gene in cardiac hypertrophy induced by isoprenalin or EPZ. Data are presented as mean ± SEM. *p < 0.05, **p < 0.01, ***p < 0.001 as compared to CON group. n = 3, experiments were repeated 3 times. **(C)** ChIP-qPCR assay showing the binding ability of Homebox A9 on three promoter regions of brain natriuretic peptide gene in cardiac hypertrophy induced by isoprenalin or EPZ. Data are presented as mean ± SEM. *p < 0.05, **p < 0.01, ***p < 0.001 as compared to CON group. n = 3, experiments were repeated 3 times. **(D)** ChIP-qPCR assay showing the binding ability of Homebox A9 and IgG (negative control) on three promoter regions of β-Myosin Heavy Chain gene. Data are presented as mean ± SEM. **p < 0.01 as compared to IgG group. n = 3, experiments were repeated 3 times. **(E)** ChIP-qPCR assay showing the binding ability of Homebox A9 on three promoter regions of β-Myosin Heavy Chain gene in cardiac hypertrophy induced by isoprenalin. Data are presented as mean ± SEM. *p < 0.05, **p < 0.01 as compared to CON group. n = 3, experiments were repeated 3 times. **(F)** ChIP-qPCR assay showing the binding ability of Homebox A9 on three promoter regions of β-Myosin Heavy Chain gene in cardiac hypertrophy induced by EPZ. Data are presented as mean ± SEM. *p < 0.05 as compared to CON group. n = 3, experiments were repeated 3 times.

Since HoxA9 participated in the anti-hypertrophic effect of PRMT5, and since HoxA9 was directly symmetric dimethylated by PRMT5, it remains unknown whether or not PRMT5 ameliorates cardiomyocyte hypertrophy through regulating HoxA9 symmetric dimethylation. HoxA9 is a transcription factor that is able to bind to promoter region of its target genes, thereby regulating the transcription of target genes (Steger et al., 2015). Thus, we explored the hypothesis that PRMT5 could regulate the binding of HoxA9 to the promotor of the potential target gene BNP and β-MHC. By conducting ChIP assay, chromatin isolated from NRCMs was enriched by HoxA9 antibody, PRMT5 antibody or normal rabbit IgG antibody (negative control) respectively, followed by Q-PCR experiments. As shown in Figure 6A, three primers were designed to amplify three different segments of BNP promoter regions. Amplification segments from all of these three primers of BNP promotor were detected in immunoprecipitate enriched by HoxA9 antibody or PRMT5 antibody, but not anti-IgG antibody, suggesting that HoxA9 and PRMT5 was able to bind to BNP promoter (Figure 6A). The binding of PRMT5 to BNP promotor was repressed in the presence of ISO and EPZ, indicating that the binding is dependent on the activity of PRMT5 (Figure 6B). In the contrary, the binding of HoxA9 to BNP promotor was enhanced when PRMT5 was suppressed or inhibited (Figure 6C). Similarly, HoxA9 was prone to binding to the promoter region of β-MHC in cardiomyocytes treated with ISO or EPZ as compared with the control (Figures 6D–F). Taken together, these results imply that PRMT5 might facilitate HoxA9 symmetric dimethylation to repress its binding to BNP and β-MHC promoter, finally abrogating BNP transcription and improving cardiomyocyte hypertrophy.

## Discussion

The PRMT family members have pivotal roles in regulating heart diseases (Beltran-Alvarez et al., 2013; Pyun et al., 2018; Onwuli et al., 2019). Among all these PRMTs, PRMT5 is an arginine methyltransferase responsible for symmetric dimethylation (Branscombe et al., 2001), and is closely involved in cardiac hypertrophy according to our previous observations (Chen et al., 2014; Cai et al., 2020). The present observations showed that silencing of PRMT5 by RNA silencing or inhibition of PRMT5 by its pharmacological inhibitor EPZ augmented the expressions of cardiomyocyte hypertrophic genes BNP and β-MHC, and that overexpression of PRMT5 inhibited ISO-induced cardiomyocyte hypertrophy. This anti-hypertrophic effect of PRMT5 is dependent on its enzymatic activity, since overexpression of the mutant PRMT5 without the methylation activity lost the ability to prevent cardiomyocyte hypertrophy. These observations are consistent with the *in vivo* studies that hearts of EPZ-treated mice were structurally larger and were demonstrated more significant hypertrophic features (Cai et al., 2020). Additionally, these results are in line with previous reports that PRMT5 protected against cardiac hypertrophy by methylating GATA4 to hamper p300-mediacted GATA acetylation (Chen et al., 2014), and by symmetric di-methylating Histone H4R3 via regulation of Filip1L/β-catenin (Cai et al., 2020). The *in vivo* and *in vitro* observations that the expression of PRMT5 was decreased in cardiac hypertrophy, imply that repression of PRMT5 might contribute to the development of cardiac hypertrophy. It also helps to explain that human with low expression of PRMT5 in peripheral blood are at high risk of coronary artery disease and acute myocardial infarction (Tan et al., 2019).

The novel finding of our study is that PRMT5 could ameliorate cardiac hypertrophy via HoxA9. Although previous studies have reported that HoxA9 was upregulated during cardiac hypertrophy (Zhou et al., 2018; Wen et al., 2019), the exact regulatory role of HoxA9 in cardiac hypertrophy remains to be elucidated. Our study confirmed that HoxA9 expression was augmented by β-adrenergic stimulation during cardiac hypertrophy. HoxA9 could promote cardiomyocyte hypertrophy, as implied by the observations that HoxA9 overexpression increased the expressions of hypertrophic genes and cardiomyocyte surface area, whereas HoxA9 knockdown prevented ISO-induced cardiomyocyte hypertrophy. Most importantly, our results demonstrated that HoxA9 could bind to the promoters of BNP and β-MHC, two typical cardiac hypertrophic markers, suggesting that HoxA9 serves as a transcription factor of BNP and β-MHC to facilitate the development of cardiac hypertrophy.

The present study reveals that HoxA9 is involved in the regulation of PRMT5 in cardiomyocyte hypertrophy. This conclusion is based on the following observations: 1) HoxA9 deficiency reversed cardiomyocyte hypertrophy induced by PRMT5 silencing or inhibition; 2) the binding of HoxA9 to BNP promoter was enhanced by PRMT5 inhibitor EPZ.

This study further investigates the mechanisms by which PRMT5 regulates HoxA9 in cardiomyocyte hypertrophy. Taken into account that PRMT5 is a symmetric dimethylase, and that PRMT5 interacted with HoxA9, it is most likely that PRMT5 directly symmetric dimethylates HoxA9. Indeed, the symmetric dimethylation level of HoxA9 was abrogated when PRMT5 was inhibited by EPZ or suppressed by ISO. These results are consistent with a previous study taken in vascular endothelial cells (Bandyopadhyay et al., 2012), supporting the conclusion that HoxA9 is a substrate of PRMT5 on symmetric dimethylation. Since EPZ or ISO augmented the binding of HoxA9 to BNP and β-MHC promoter, it is probably that symmetric dimethylation of HoxA9 by PRMT5 might impair its promoter binding activity, ultimately repressing the transcription of BNP or/and other hypertrophic genes.

Intriguingly, PRMT5 affects the expression of HoxA9 at the transcriptional level. This is based on the observations that si-PRMT5 augmented HoxA9 expression while PRMT5 overexpression decreased HoxA9 protein level, and that knockdown or inhibition of PRMT5 increased the mRNA level of HoxA9. These results imply that PRMT5 suppresses HoxA9 through histone methylation as an epigenetic modification mechanism, since PRMT5 is a vital histone methyltransferase catalyzing symmetric dimethylation in histones H4R3 and H3R8 (Zhao et al., 2009; Saha et al., 2016). By methylating these histones, the transcription of target genes might probably be repressed. Another possible explanation is that the symmetric dimethylation of HoxA9 induced by PRMT5 might possibly affect its ubiquitination. Indeed, PRMT5 is reported to induce ubiquitination of Dual-specificity phosphatase 14 (DUSP14) by arginine methylation (Yang et al., 2018). This might support the hypothesis that there is crosstalk between ubiquitination and methylation of HoxA9, and that HoxA9 methylation induced by PRMT5 might facilitate its ubiquitination degradation. Further investigations will be required to unveil the mechanisms underlying regulation of PRMT5 on HoxA9.

In conclusions, PRMT5 exerts potent anti-hypertrophic effect by symmetric dimethylating HoxA9 and repressing HoxA9 expression. These findings suggest that activation of PRMT5 or inhibition of HoxA9 might serve as potential therapeutic strategies in treatment of cardiac hypertrophy.

## Data Availability Statement

The original contributions presented in the study are included in the article/Supplementary Material, further inquiries can be directed to the corresponding authors.

## Ethics Statement

The animal study was reviewed and approved by Institutional Animal Care and Use Committee (IACUC), Sun Yat-Sen University.

## Author Contributions

SC and RL designed and performed research, analyzed data and wrote the paper; PW and JL contributed to new reagents, analytic tools and experimental protocols; TX contributed to data collections. MW and YC contributed to cell culture and preparations for sample inspection. PL and ZL helped provide constructive informations to the framework of this manuscript and contributed to refine the main idea of this article.

## Funding

This work was supported by grants from the National Natural Science Foundation of China (81872860, 81673433, 81973318), Local Innovative and Research Teams Project of Guangdong Pearl River Talents Program (2017BT01Y093), National Major Special Projects for the Creation and Manufacture of New Drugs (2019ZX09301104), Special Program for Applied Science and Technology of Guangdong Province (2015B020232009), National Engineering and Technology Research Center for New drug Druggability Evaluation (Seed Program of Guangdong Province, 2017B090903004), Guangzhou Science and Technology Program Project (201604020121, 201804010227), Yang Fan Project of Guangdong Province (Grant no. 2014 YT02S044), and Guangdong Provincial Key Laboratory of Construction Foundation (No. 2017B030314030).

## Conflict of Interest

The authors declare that the research was conducted in the absence of any commercial or financial relationships that could be construed as a potential conflict of interest.
