# Supplementary material for: PRMT5 Prevents Cardiomyocyte Hypertrophy via Symmetric Dimethylating HoxA9 and Repressing HoxA9 Expression
Source: Front Pharmacol. 2020 Dec 10;11:600627. doi: 10.3389/fphar.2020.600627 (PMC7793800; doi:10.3389/fphar.2020.600627)
Supplement: Supplementary file 1 [file datasheet1.docx]

**Table S1. Primers of target genes used in quantitative polymerase chain reactions (Q-PCR)**

| **Gene** | **Sequences** |
| --- | --- |
| **PRMT5** | **Forward: 5’-TCACTCTGAGTATCCGCCCA-3’** |
|  | **Reverse: 5’-CCCACTCGTACCACACCTTC-3’** |
| **HoxA9** | **Forward:5'-CCCTCAGCAAGACGAACTCC-3'** |
|  | **Reverse:5'-GGAACTGCCAAGGAGAGGAC-3'** |
| **ANF** | **Forward:5'-CTGCTAGACCACCTGGAGGAGAAG-3'** |
|  | **Reverse:5'-TCATCGGTCTGCTCGCTCAGG-3'** |
| **BNP** | **Forward:5'-AGTCTCCAGAACAATCCACGATGC-3'** |
|  | **Reverse:5'-CCGGAAGGCGCTGTCTTGAG-3'** |
| **β-MHC** | **Forward:5'-GTGCCAATGACGACCTGAAGGAG-3'** |
|  | **Reverse:5'-CTGGTTGATGAGGCTGGTGTTCTG-3'** |
| **β-actin** | **Forward:5’- ACAACCTTCTTGCAGCTCCTC-3’** |
|  | **Reverse:5'-CTGACCCATACCCACCATCAC-3'** |

**Table S2. Primers of different promoter region located in BNP**

| **Gene** | **Sequences** |
| --- | --- |
| **BNP-region1** | **Forward:5'-GCTCTAAAGCACCTCAGTCACCT-3'** |
|  | **Reverse:5'-CCATCAGGAAGCAGAGAGCAGTG-3'** |
| **BNP-region2** | **Forward:5'-ATGGCTTCAAGCTCCTGCCAC-3'** |
|  | **Reverse:5'-CCCAGCTTCATTCTTAGCTGCAGC-3'** |
| **BNP-region3** | **Forward:5'-GAGTGTGTCTCCCTTGGGTCAG-3'** |
|  | **Reverse:5'-CCCACTCAGGTGAGAAATGAGACTCT-3'** |
|  |  |

**Table S3. Sequence of target genes specifically identified by small interference RNAs**

| **Gene** | **Targeted sequence** |
| --- | --- |
| **PRMT5** | **GCTAATTGTGGGAAAGCTT** |
| **HoxA9** | **TGTATCACCACCACCACCA** |
